# A case study of combined neoadjuvant chemotherapy and neoadjuvant immunotherapy in resectable locally advanced esophageal cancer

**DOI:** 10.1186/s12957-022-02732-w

**Published:** 2022-08-26

**Authors:** Huiru Dai, Minling Liu, Xueying Li, Tingwei Li, Wensheng Huang, Jiehao Liao, Yun Li, Shuo Fang

**Affiliations:** 1 The Department of Clinical Oncology, Guangdong Provincial Key Laboratory of Digestive Cancer Research, Big data Centre, The Seventh Affiliated Hospital, Sun Yat-Sen University, Shenzhen, Guangdong 518107 People’s Republic of China; 2 The Department of Radiology, The Seventh Affiliated Hospital, Sun Yat-Sen University, Shenzhen, Guangdong 518107 People’s Republic of China; 3 The Department of Thoracic Surgery, The Seventh Affiliated Hospital, Sun Yat-Sen University, Shenzhen, Guangdong 518107 People’s Republic of China

**Keywords:** Neoadjuvant therapy, Immunotherapy, PD-1 inhibitor, Esophageal cancer

## Abstract

**Background:**

The prognosis of patients under existing neoadjuvant chemotherapy or neoadjuvant chemoradiotherapy requires improvement. Whereas programmed cell death 1 (PD-1) inhibitors have shown promising response in advanced esophageal cancer, they have not been used in the perioperative treatment of resectable locally advanced esophageal cancer. Whether immunotherapy can be incorporated into neoadjuvant therapy has became a challenging question for researchers.

**Case presentation:**

We present a case of a 65-year-old male who had a history of progressive dysphagia for approximately 1 month. He underwent pertinent studies including computed tomography (CT)，gastroscopy，and pathological biopsy resulting in a diagnosis of medium-low differentiated squamous carcinoma of the thoracic segment of the esophagus (cT2N2M0 stage III). After 4 cycles of neoadjuvant chemotherapy combined with immunotherapy, gastroscopy showed the lesion in the esophagus was no longer present. Subsequently, the patient received thoracoscopic radical resection of esophageal cancer and achieved a pathological complete response (pCR) in postoperative pathological evaluation. During the whole treatment, no adverse effect was recorded and to date no evidence of recurrence has been recorded.

**Conclusion:**

Our report suggest that neoadjuvant chemotherapy combined with immunotherapy not only improve the R0 resection and pCR rate in patients with resectable locally advanced esophageal cancer, but also the adverse effects are within the control range. However, the selection of therapeutic strategy, predictors of response to treatment, and interval time between neoadjuvant treatment and surgery still await more reliable evidence-based studies with large prospective samples.

**Supplementary Information:**

The online version contains supplementary material available at 10.1186/s12957-022-02732-w.

## Background

Esophageal cancer is the seventh most common cancer worldwide, and has the sixth highest mortality rate among all malignancies [1]. Esophageal cancer is divided pathologically into two major types: adenocarcinoma and esophageal squamous cell carcinoma (ESCC), of which ESCC is more prevalent in Asian countries, especially in China [2]. Despite the development of multidisciplinary treatments such as surgery, radiotherapy, and chemotherapy, the prognosis of patients with esophageal cancer is still unsatisfactory.

Immune checkpoints enhance the tumor-killing function of immune cells by relieving the body's inhibitory effect on immune cells. Many clinical trials have been initiated to explore the immunotherapy of esophageal cancer [3], and immune checkpoint inhibitors (ICI) have been approved for The National Comprehensive Cancer Network (NCCN)/Chinese Society of Clinical Oncology (CSCO) first-line，second line and postoperative adjuvant therapy for esophageal cancer [4–7]. However, no reliable clinical studies showed the benefit of neoadjuvant immunotherapy in patients with esophageal cancer.

Neoadjuvant therapy refers to certain treatments that are given before radical treatment. It is used to improve the surgical R0 resection rate by reducing the tumor volume, size, or number of lymph nodes, and to reduce the recurrence rate by eliminating micrometastasis [8, 9]. In resectable locally advanced esophageal cancer, according to CROSS study [10], neoadjuvant chemoradiotherapy is approved as the standard treatment in western countries. While in Japan, perioperative neoadjuvant chemotherapy is preferred because of the positive results of the JCOG9907 study [11]. However, the 5-year recurrence rate is still as high as 40–50% [12]. This may be partly due to insufficient systemic therapy at either preoperative or postoperative period. For postoperative period, researches have confirmed only selected patients benefited from adjuvant therapy in clinical trails [13], and others enter surveillance until progression. So, enhancing preoperative treatments maybe a potential way to reduce the recurrence rate.

Neoadjuvant immunotherapy can induce a stronger immunological effect on tumor cells at an early stage of cancer by inducing T cell expansion with less impaired T-cell function compared with that induced by postoperative adjuvant immunotherapy [14–16]. In recent years, with approval of immunotherapy in the first line，second line treatment and postoperative adjuvant therapy, researchers have begun to study its efficacy in the perioperative period, including its positive impact on increasing surgical resection rates and improving long-term prognosis. Small sample clinical trials are already underway [17, 18], but have yet to confirm their reliability. In the present study, we report a case of a patient with locally advanced squamous esophageal cancer who underwent 4 cycles of immunotherapy combined with chemotherapy in the preoperative period and achieved a pCR on preoperative evaluation.

## Case presentation

**Fig. 1:**
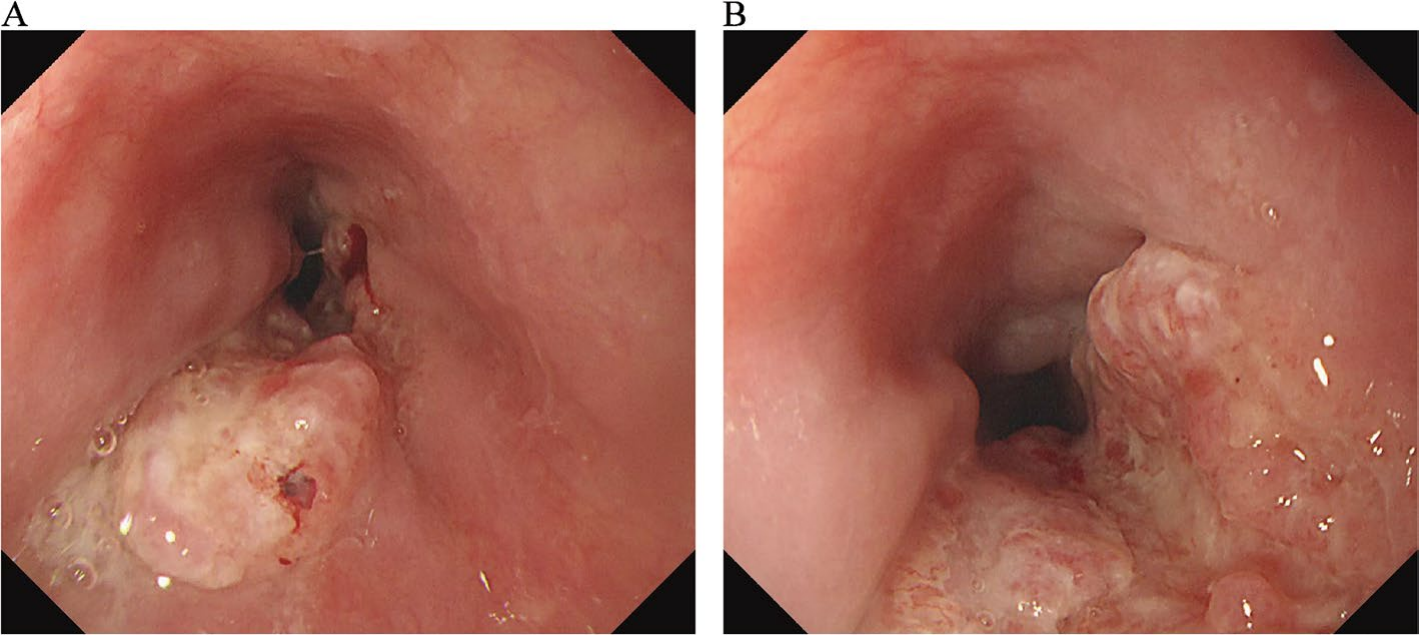
Pre-treatment examination: Gastroscopic findings (**A**, **B**)

**Fig. 2:**
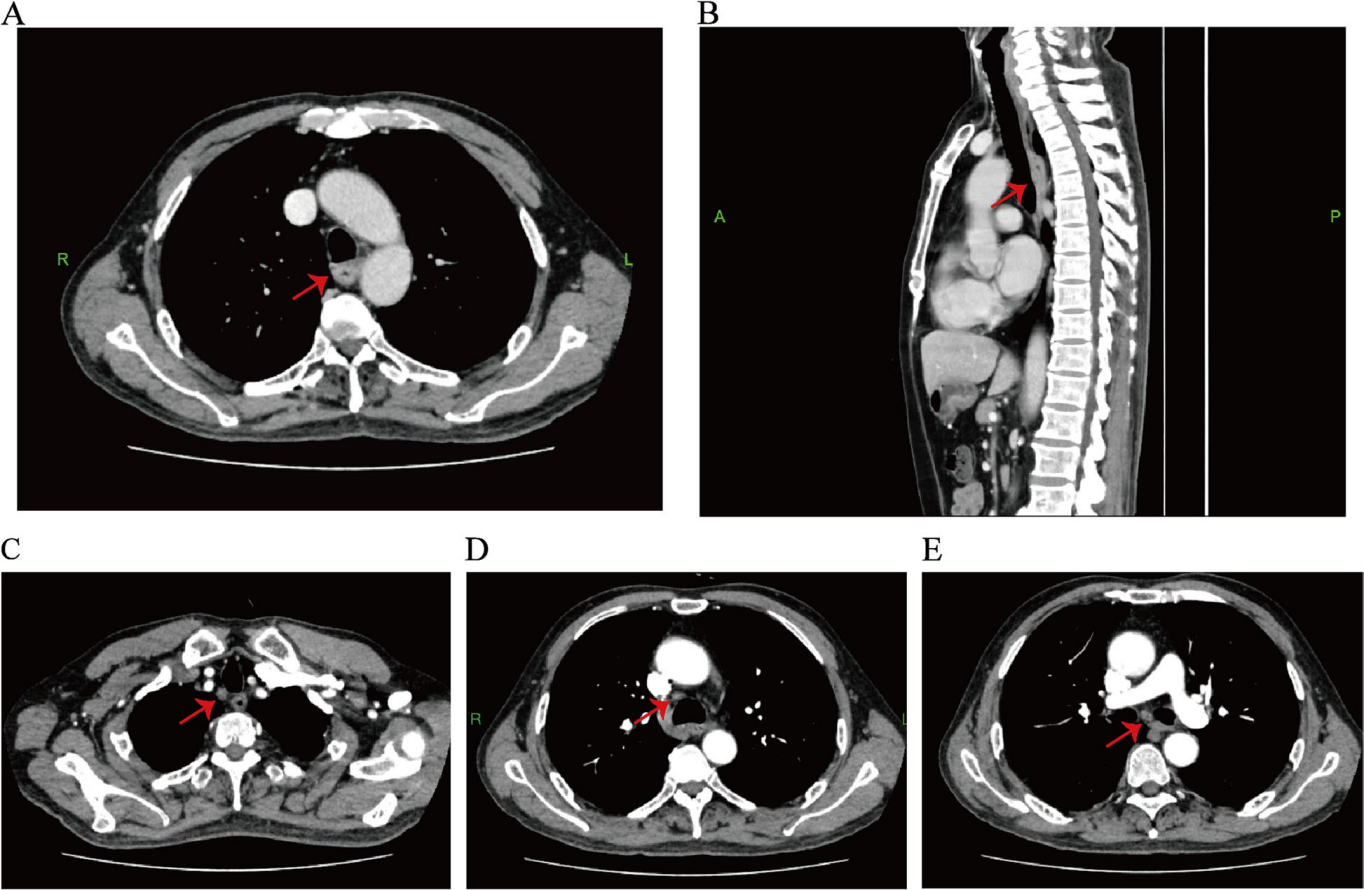
Pre-treatment examination: computed tomography. **A**, **B** The red arrows indicate the esophageal cancer lesions. **C**–**E** The red arrows indicate the swollen lymph nodes

**Fig. 3:**
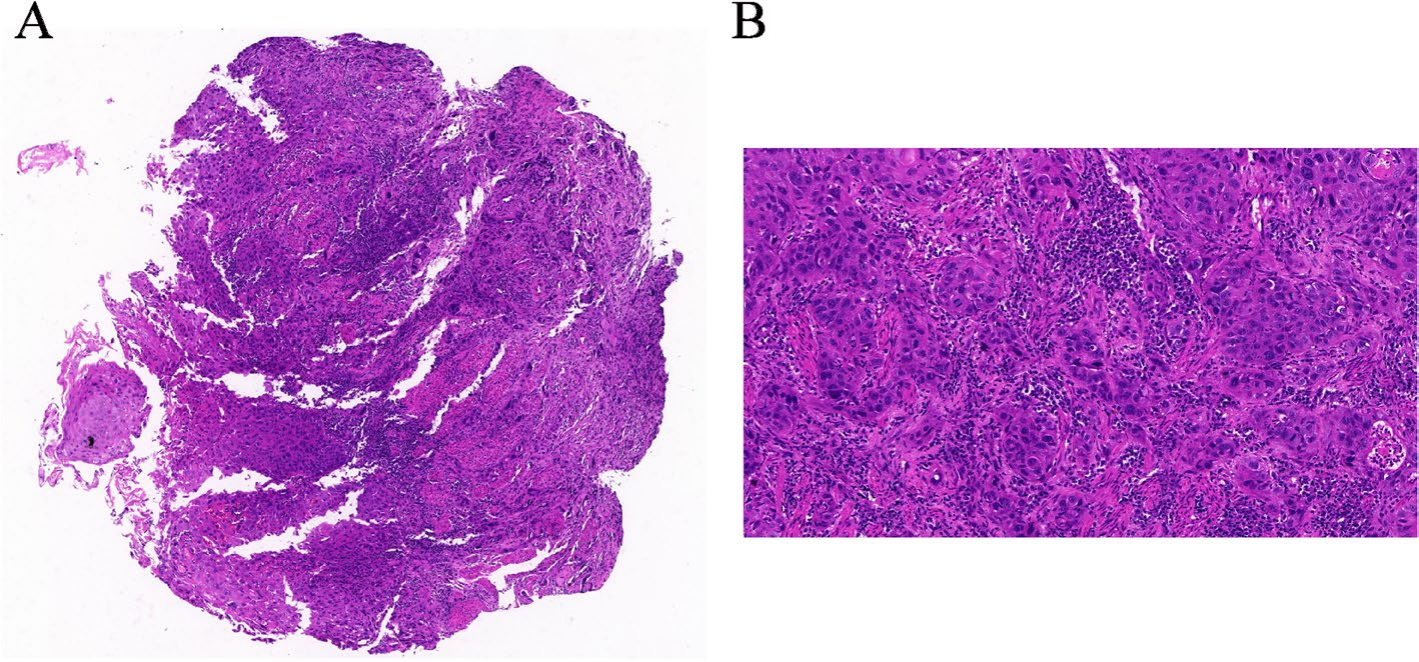
Pre-treatment examination: histopathological findings (**A**, **B**). The images were obtained after H&E staining under a magnification of respectively 50× and 200×

**Fig. 4:**
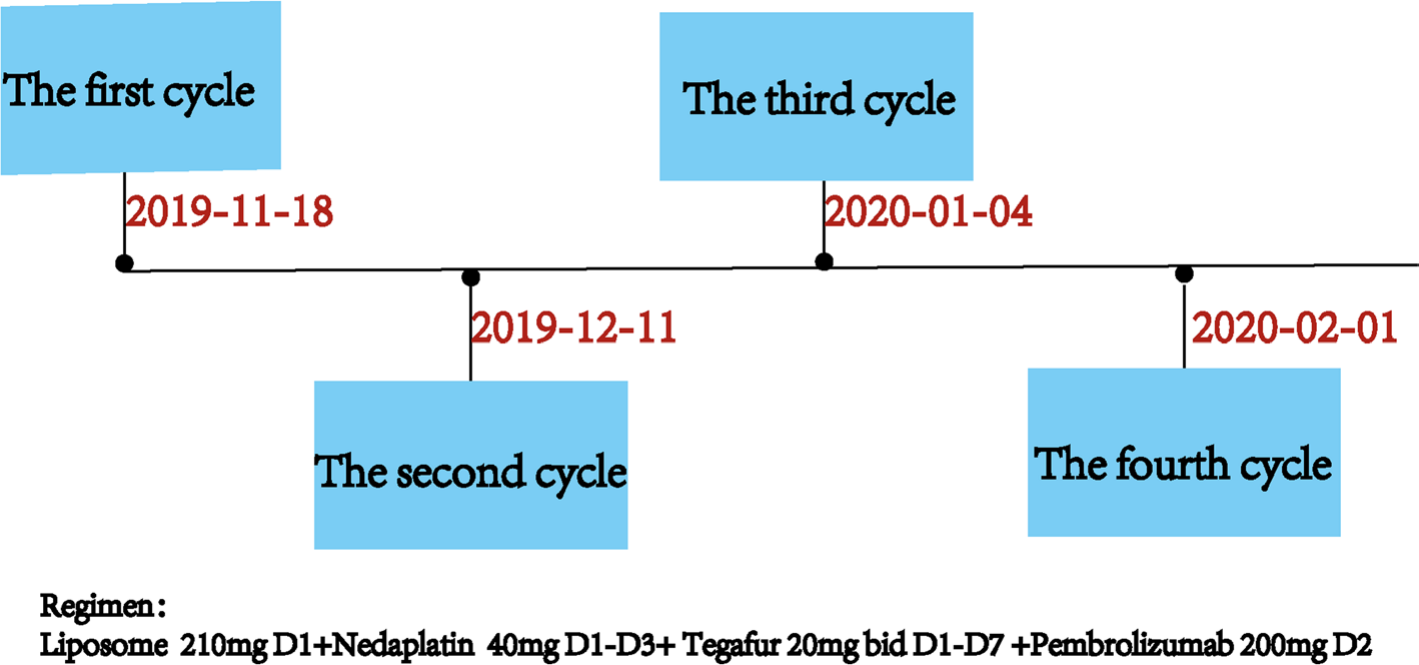
The doses and schedules of the neoadjuvant chemotherapy combined neoadjuvant immunotherapy

**Fig. 5:**
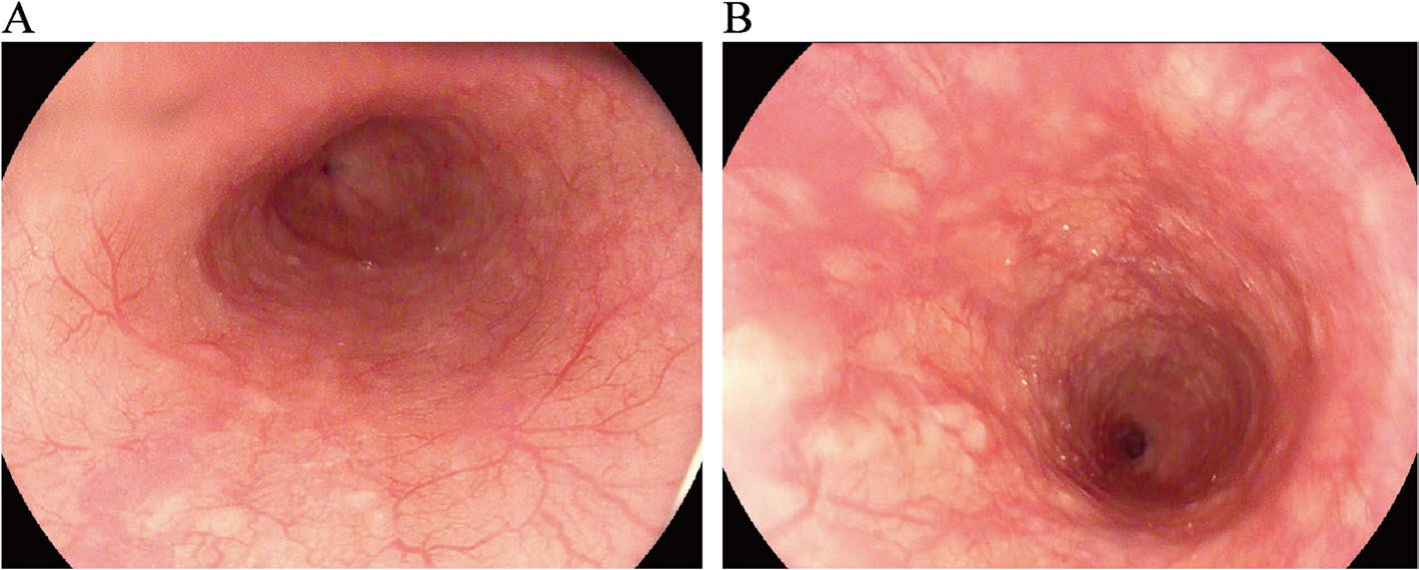
Post-treatment examination: gastroscopic findings (**A**, **B**), respectively middle esophagus and lower esophagus

**Fig. 6:**
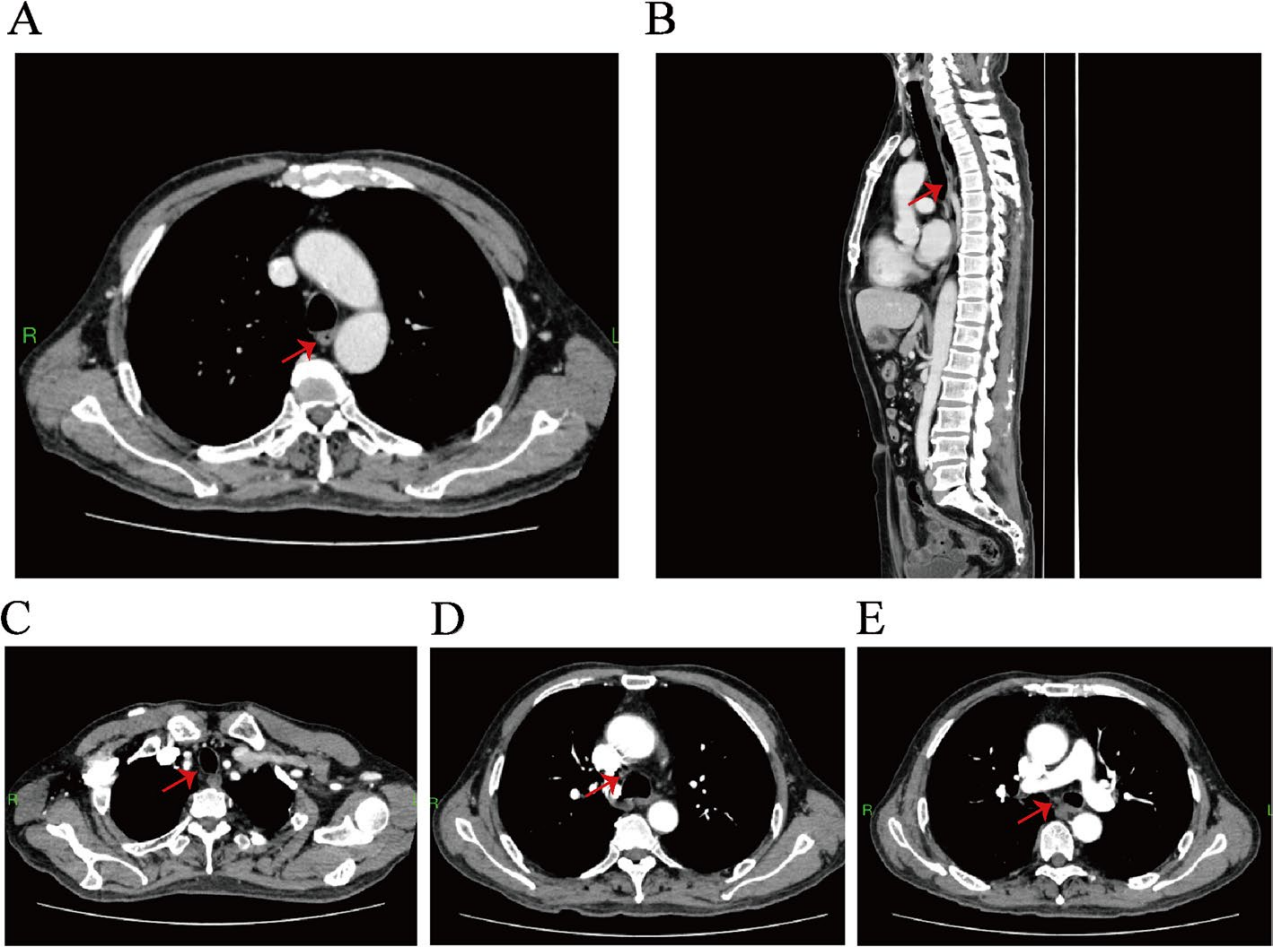
Post-treatment examination: computed tomography (**A**, **B**). The red arrows indicate the esophageal cancer lesions. **C**–**E** The red arrows indicate lymph nodes. All of them became smaller compared with pre-treatment

**Fig. 7:**
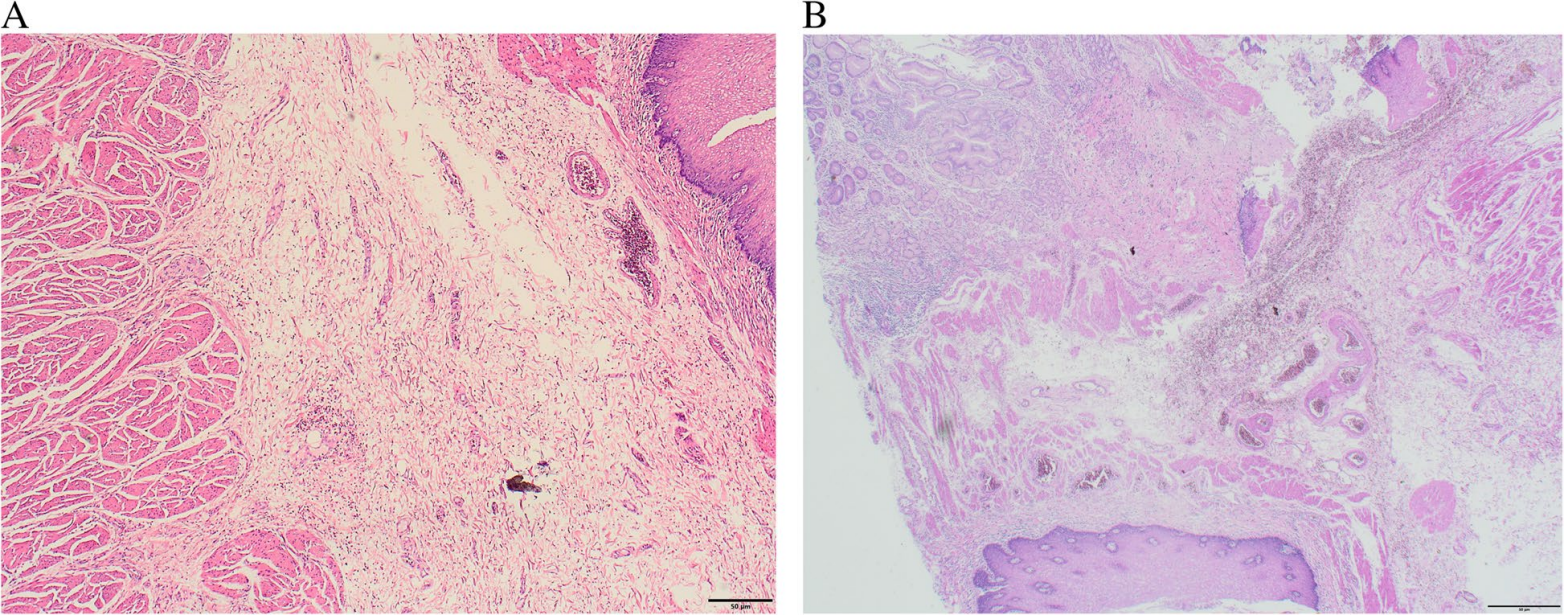
Post-treatment examination: postoperative histopathological findings (**A**, **B**). The images were obtained after H&E staining under a magnification of respectively 40× and 40×. The squamous epithelium is intact, and subepithelial proliferation of fibrous connective tissue with vasodilatation and inflammatory cell infiltration

To protect patient's privacy, important patient information is withheld in the following description. The patient is a 65-year-old male with a 5-year history of previous coronary artery disease, who was implanted with a stent in 2016, 2017, and 2019, respectively, and has been taking aspirin, ticagrelor, and atorvastatin postoperatively. The patient denied a history of smoking and alcohol consumption. He was admitted to a hospital after suffering from progressive dysphagia for about 1 month, and was subjected to a gastroscopy, which showed an irregular bulge in the esophageal wall at 22~26 cm from the incisor, occupying 50% of the intestinal lumen (Fig. 1A, B). He was further referred to our hospital. The admission evaluation Eastern Cooperative Oncology Group (ECOG) Performance Status (PS) assessment was 1 point. Computed tomography (CT) of the chest + whole abdomen showed: (1) uneven thickening of the mid-thoracic wall of the esophagus, consistent with esophageal cancer, with a thickness of about 15 mm and a length of about 38 mm (Fig. 2A, B); (2) multiple swollen lymph nodes in the mediastinum, indicating metastasis, the larger one being 10 mm × 10 mm (Fig. 2C–E). Gastroscopy pathological biopsy (Fig. 3A, B) taken from the above hospital and further performed in our hospital showed that esophagus tumor cells showed single scattered or nest-like infiltrative growth, rich cytoplasm, red staining, localized keratinization, which was consistent with medium-low differentiation squamous cell carcinoma. Combined with the above examination, the patient was diagnosed as having (1) medium-low differentiated squamous carcinoma of the thoracic segment of the esophagus (cT2N2M0, stage III, according to the 8th [2017] edition of the American Joint Committee on Cancer staging system); (2) coronary artery disease; stable angina pectoris; and cardiac function class II status after coronary artery stent implantation. After obtaining the patient’s informed consent, four cycles of neoadjuvant chemotherapy combined neoadjuvant immunotherapy (Liposome 210 mg D1 + Nedaplatin 40 mg D1-3 + Tegafur 20 mg bid D1-7 + Pembrolizumab 200 mg D2) were performed from 18 November 2019 to 01 February 2020, 3 weeks a cycle (Fig. 4). After the fourth cycle, gastroscopy (Fig. 5A, B) showed that the lesion at 22~26 cm from the incisor in the esophagus was no longer present and local scar-like changes were also not obvious. Additionally, the patient received CT (Fig. 6A–E) and achieved CR (complete response) assessed by Response Evaluation Criteria in Solid Tumors (RECIST) V.1.1 [19]. On 16 March 2020, thoracoscopic radical resection of esophageal cancer was performed, and the procedure went smoothly. Postoperative pathological biopsy (Fig. 7A, B) showed (1) no clear cancer residue was seen in lymph nodes of groups 7, 8, 8a, 10L, and 12P; (2) no clear cancer residue was seen in all esophageal tissues sent for examination; (3) the tumor regression grading (TRG) was grade 0 according to guidelines of College of American Pathologists [20]. The whole treatment was well tolerated by the patient, and no adverse effects were observed during treatment. As of the date of publication of this article, the patient was still in CR status and PFS had not yet been achieved.

## Discussion

Although neoadjuvant chemotherapy or chemoradiotherapy combined with surgery helps to improve resection rate and survival in patients with locally advanced esophageal cancer, surgical R0 resection rate remains poor and most patients present with a recurrence pattern dominated by distant metastases. According to Shapiro et al., this recurrence pattern might result from insufficient intensity of systemic therapy [12]. Therefore，activation of immunity, a systemic therapy, might help to improve the failure pattern of high distant metastasis rate after the original neoadjuvant therapy. Based on the above theory, for the case staged as cT2N2M0, we performed neoadjuvant chemotherapy and added immunotherapy as a way to enhance systemic therapy. The patient achieved R0 resection, and there has been no recurrence so far.

Based on the case report, our article raises following questions that remain to be addressed.

Firstly, identification of metastatic lymph nodes. For patients diagnosed as locally advanced esophageal carcinoma without distant metastasis, the surgical approach and the potential application of neoadjuvant therapy are dependent on preoperative stage, especially N stage. However, 11~56% of lymph nodes were reported to falsely diagnosed as negative for metastasis [21]. Traditionally, only lymph nodes with short diameters > 10 mm, roundish and heterogenous internal density would be considered as metastasis. Nevertheless, Wakita et al. found that [22] 89% of the false negative lymph nodes did not exceed 8 mm in size. In our case, the diameter and shape of one lymph node was also not typical, but under the guidance of our experienced imaging expert, we identified the lymph node. For the vital issue above, maybe more explorations are needed to accurately identify metastatic lymph nodes.

Secondly, the new-emerging “neoadjuvant immunotherapy”, whether should it be used in combination with chemotherapy or chemoradiotherapy. Researches comparing two current and standard neoadjuvant therapy “chemotherapy and neoadjuvant chemoradiotherapy” in resectable locally advanced esophageal carcinoma have revealed no convincing evidence and unified conclusion [23–25]. For example, a retrospective analysis conducted by Koch showed clear advantage in overall survival for neoadjuvant chemoradiotherapy in comparison with chemotherapy [23], while Yamagata suggested that chemotherapy might be more effective than neoadjuvant chemoradiotherapy [24]. So，these two therapy strategies are still practiced according to respective institutional protocol. As for the new-emerging therapeutic strategy “neoadjuvant immunotherapy”, whether should it be used in combination with chemotherapy or chemoradiotherapy remains a significant question. ①Immunotherapy combined with chemotherapy. Chemotherapy can further activate the immune system by promoting tumor antigen presentation and destroying immunosuppressive factors [26, 27]. A pilot study combining immunotherapy with chemotherapy reported a satisfactory pCR rate of 31.3%, R0 resection rate of 93.8% and acceptable tolerance in locally advanced ESCC patients [28]. Similar result was also found in our case. ② Immunotherapy combined with chemoradiotherapy. The tumor microenvironment (TME) created by chemoradiotherapy might increase sensitivity to immunotherapy and promote anti-tumor immune response. One study has confirmed that overall survival in ICI combined with chemoradiotherapy was higher than that in the group of concurrent chemoradiotherapy without immunotherapy [17]. Nowadays，clinical trials comparing these two new emerging therapeutic strategies are underway. Therefore, typical application of neoadjuvant treatment in clinical case, would open new way and provide practical evidence for cancer treatment [29, 30].

Thirdly, selection of predictors for response to neoadjuvant immunotherapy. Some patients might exist as neoadjuvant therapy nonresponders, leading to tumor progression and delaying optimal treatment. Consequently, it is vital to screen the responding population with predictors. For example, for neoadjuvant chemotherapy, serum IgG level was found to be a satisfactory predictor [31]. For immunotherapy, researchers have focused on several biomarkers, mainly PD-L1 [32–35], the tumor mutation burden (TMB) [36–38], Mismatch repair (MMR) status [39], and tumor-infiltrating lymphocytes (TILs) [40–42]. In summary, PD-L1, TMB, MMR status, or TILs singly might not accurately predict the effect of immunotherapy, and whether to combine multiple predictors in neoadjuvant immunotherapy remains an outstanding question [43]. The case discussed in our study was not tested for these indicators before and after treatment, which is a shortcoming. If those expression could be characterized or quantified before treatment, they might provide solid evidence on predicting efficacy of neoadjuvant immunotherapy combined with chemotherapy.

Finally, the optimal time interval between neoadjuvant therapy and surgery is also concerned. In clinical trials, the standard time to surgery (TTS) after completed neoadjuvant chemoradiotherapy is 4–6 weeks. However, due to administration reasons or being not suitable for surgery, a significant number of patients underwent prolonged TTS after neoadjuvant therapy. Kuo has focused on such an issue concerning survival between standard TTS and prolonged TTS, and has detected no significant difference [44]. For the new emerging therapeutic strategy“neoadjuvant immunotherapy combined with chemotherapy or chemoradiotherapy”, we presented a TTS of 6 weeks. The optimal TTS still remains to be explored. Furthermore, more clinical trials should be performed to clarify differences in clinical benefit between TTS and prolonged TTS.

## Conclusion

In resectable locally advanced esophageal cancer, neoadjuvant chemotherapy or chemoradiotherapy combined with surgery has been used as the standard treatment; however, its therapeutic efficacy still needs to be improved. Although neoadjuvant immunotherapy is still at the stage of exploration in small samples, the available clinical data show that its efficacy is significant and its safety and adverse effects are within the control range. In future, neoadjuvant immunotherapy is expected to provide a new treatment strategy for patients with locally advanced esophageal cancer. In addition, based on clinical trial of large prospective samples, the selection of therapeutic strategy, predictors of response to treatment, and interval time between neoadjuvant treatment and surgery will be the essential research directions of neoadjuvant therapy.

## Supplementary Information

**Additional file 1.**

**Additional file 2.**

## Data Availability

All the data are available in the patient’s medical record.

## Abbreviations

ESCC: Esophageal squamous cell carcinoma
PD-1: Programmed cell death 1
ICIs: Immune checkpoint inhibitors
pCR: Pathological complete response
TRG: Tumor regression grading
ECOG: Eastern Cooperative Oncology Group
PS: Performance status
CT: Computed tomography
NCCN: National Comprehensive Cancer Network
CSCO: Chinese Society of Clinical Oncology
TME: Tumor microenvironment
TMB: The tumor mutation burden
MMR: Mismatch repair
TILs: Tumor-infiltrating lymphocytes
MSI-H: Microsatellite instability-high
