# Supplementary figures and images for: A case study of combined neoadjuvant chemotherapy and neoadjuvant immunotherapy in resectable locally advanced esophageal cancer
Source: World J Surg Oncol. 2022 Aug 26;20:267. doi: 10.1186/s12957-022-02732-w (PMC9414113; doi:10.1186/s12957-022-02732-w)

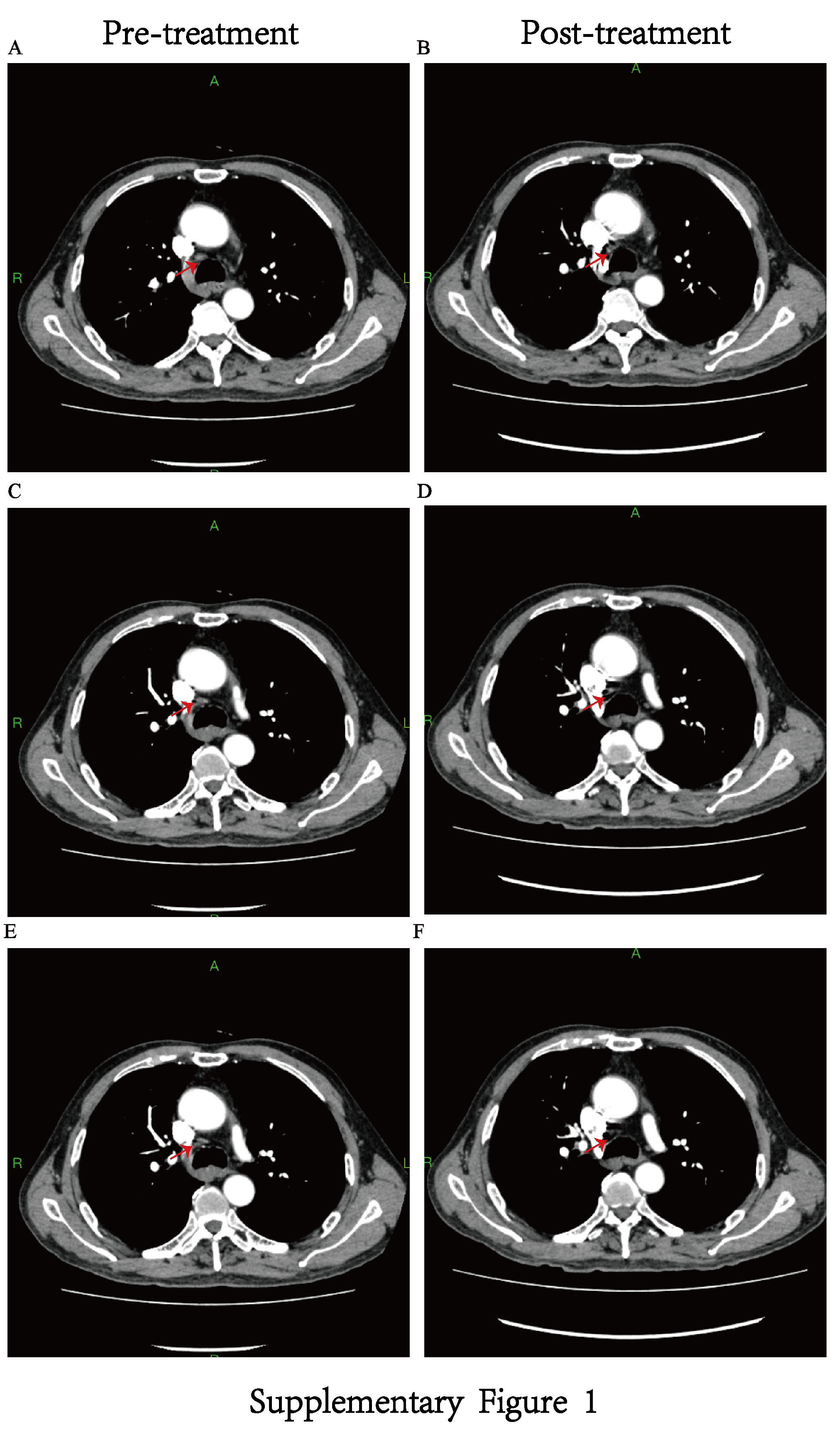

Supplement: Supplementary file 1 — Additional file 1. [file 12957_2022_2732_MOESM1_ESM.tif]

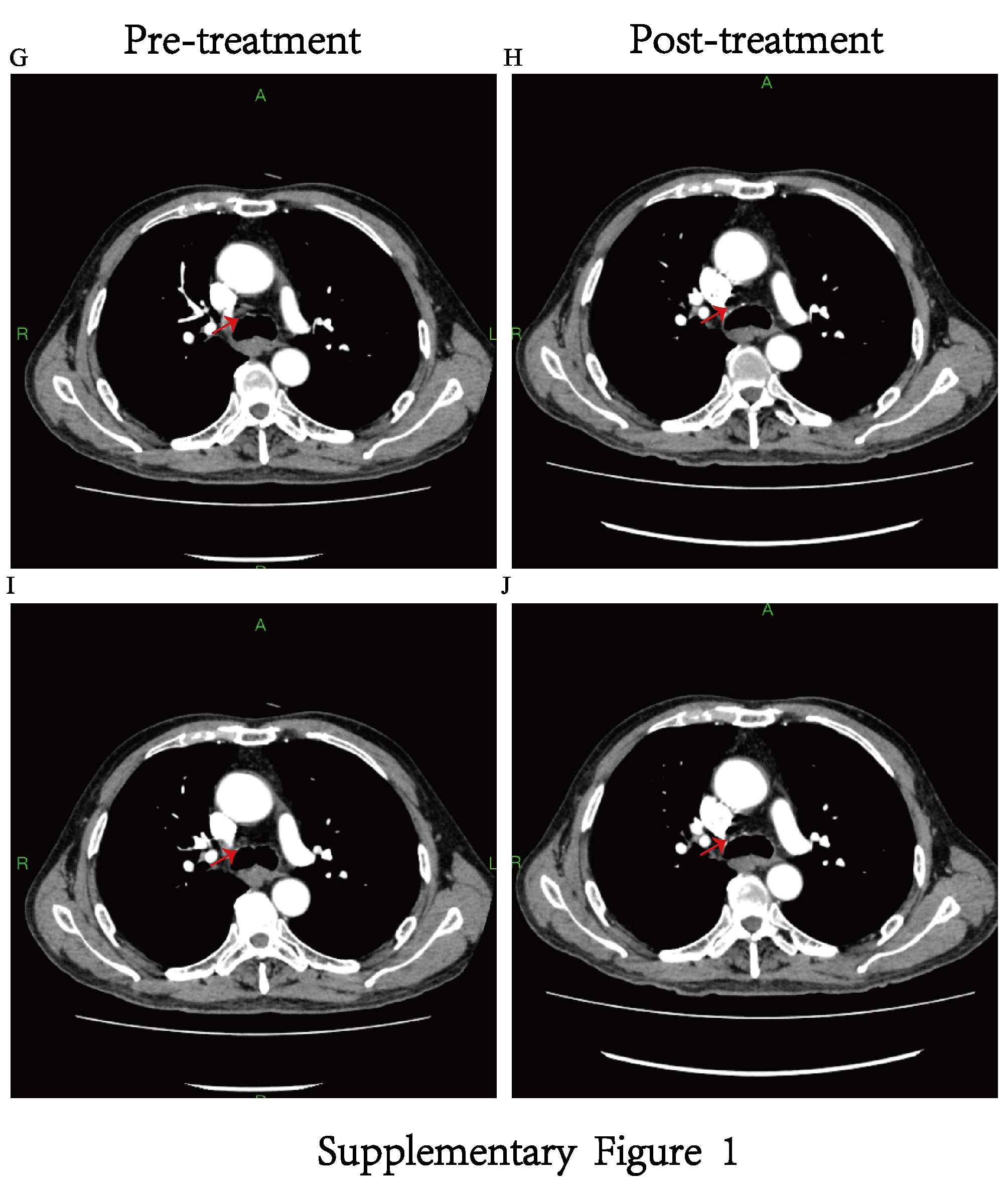

Supplement: Supplementary file 2 — Additional file 2. [file 12957_2022_2732_MOESM2_ESM.tif]
